# How Foliar Fungal Diseases Affect Nitrogen Dynamics, Milling, and End-Use Quality of Wheat

**DOI:** 10.3389/fpls.2020.569401

**Published:** 2020-11-19

**Authors:** María Rosa Simón, María Constanza Fleitas, Ana Carolina Castro, Matías Schierenbeck

**Affiliations:** ^1^Cerealicultura, Facultad de Ciencias Agrarias y Forestales, Universidad Nacional de La Plata, La Plata, Argentina; ^2^Comisión de Investigaciones Científicas Provincia Buenos Aires, La Plata, Argentina; ^3^Department of Plant Sciences, College of Agriculture and Bioresources, University of Saskatchewan, Saskatoon, SK, Canada; ^4^Consejo Nacional de Investigaciones Científicas y Técnicas, La Plata, Argentina; ^5^Genebank Department, Leibniz Institute of Plant Genetics and Crop Plant Research (IPK), Gatersleben, Germany

**Keywords:** fungal pathogens, foliar disease severity, fungicides, N fertilization, N remobilization, N post-anthesis absorption, bread-making quality, bread wheat

## Abstract

Foliar fungal diseases may cause important losses on yield and quality of wheat (*Triticum aestivum* L.). They may impact crop growth rate differently, modifying nitrogen (N) dynamics and carbohydrate accumulation in the grain. The relationship between N and carbohydrates accumulation determines the grain protein concentration, which impacts the gluten concentration and rheological properties of the wheat flour. In addition, types of fungicides and N fertilization can influence the intensity of foliar diseases and have an effect on the milling and end-use quality, depending on the bread-making aptitude of the genotypes, the nutritional habit of the pathogen involved, the amount and time of infection, environmental factors, and interactions between these factors. In that way, N fertilization may modify the severity of the diseases according to the nutritional habit of the pathogen involved. Some fungicides, such as strobilurins and carboxamides, produce high levels of disease control and prolong the healthy leaf area duration, which translates into important yield responses, potentially compromising the grain protein concentration by additional carbohydrate production, with consequences in the bread-making quality. Furthermore, infections caused by biotrophic pathogens can be more damaging to N deposition than to dry matter accumulation, whereas the reverse has been generally true for diseases caused by necrotrophic pathogens. The time of infection could also affect yield components and N dynamics differentially. Early epidemics may reduce the number of grains per area and the N remobilization, whereas late epidemics may affect the thousand kernel weight and mainly the N absorption post-flowering. A review updating findings of the effects of infections caused by foliar fungal pathogens of different nutritional habits and the incidence of several factors modifying these effects on the above-ground biomass generation, N dynamics, protein and gluten concentration, milling, rheological properties, loaf volume, and other quality-related traits is summarized. Three main pathogens in particular, for which recent information is available, were taken as representative of biotrophic (*Puccinia triticina*), necrotrophic (*Pyrenophora tritici*-*repentis*), and hemibiotrophic (*Zymoseptoria tritici*) nutritional habit, and some general models of their effects are proposed. New challenges for researchers to minimize the impact of foliar diseases on end-use quality are also discussed.

## Introduction

Meeting the growing demand for food and increasing the nutritional quality of crops over the next 30 years will be challenging, given the rapid population growth (FAO, IFAD, UNICEF, WFP, and WHO, 2019). Bread wheat (*Triticum aestivum* L.) is an important portion of the standard diet for many people in most countries where it is consumed regularly as a main source of calories. Wheat also provides a number of components that are essential for health, notably protein, vitamins (mainly B vitamins), dietary fiber, and phytochemicals (Shewry and Hey, 2015). Yield potential and attainable yield in wheat, which are affected by biotic and abiotic stresses, must dramatically increase in future years to meet the forecasted world demand; this increase should be accompanied by improved nutritional quality.

Wheat quality is a complex concept. It is defined in terms that represent value to a specific end-user, i.e., wheat quality is perceived differently, depending on the stakeholders of the wheat value chain. Farmers will value a wheat variety that produces high yields and allows them to allocate the harvested grain at the highest price in the market, whereas millers will focus on getting high flour yields during milling. For their part, manufacturers will put emphasis on (i) processing quality, which is the aptitude of a particular variety to be processed at a low cost to obtain a constant result, and (ii) the end-use quality, which is the ability to produce a specific product that meets consumer requirements. For millers, grain hardness and density (test weight) are important parameters of milling quality (Guzmán et al., 2016), whereas for processing as well as end-use quality, grain hardness, grain protein concentration (GPC), gluten quality, and quantity affecting rheological properties are important. Furthermore, nutritional quality, which is important for physical health, is becoming a priority for food producers (Köpke, 2005).

The main components of the endosperm of grain wheat are starch and protein. Starch, which is composed of amylose and amylopectin, is the key factor determining wheat yield, accounting for 65 to more than 80% of grain weight (Hurkman et al., 2003). The starch physicochemical properties are influenced by the ratio of amylose to amylopectin (about 25–30% to 70–75%) which are essential for the end-use quality. Carbohydrates in the grains mainly come from photosynthesis during the grain filling (Blum, 1998). The other important storage compound of the wheat grain is protein-related with the nitrogen (N) dynamics and accounts for 10–15%, whose composition is essential for flour quality (Li et al., 2018). The deposition and redistribution of N are crucial processes regulating grain yield and grain quality in wheat (Gaju et al., 2014). High uptakes of N are critical for obtaining high grain yields or high grain quality (Barraclough et al., 2014). Nitrogen grain yield is mainly derived from (i) the amount of N accumulated in the plant previous to anthesis and remobilized to the filling grains (N remobilization; NREM); (ii) the N uptake from the soil after anthesis (N post-anthesis absorption; NPA), and (iii) the redistribution during grain development (Masclaux-Daubresse et al., 2010; Gaju et al., 2014). An important amount of N in mature grains (50–95%) comes from the NREM (Palta and Fillery, 1995; Kichey et al., 2007), the main sources of which are stems and leaves (Critchley, 2001). Furthermore, NPA can provide between 5 and 50% of grain N (Van Sanford and MacKown, 1987; de Ruiter and Brooking, 1994), depending on the N available in the soil, environmental conditions, and the effect of abiotic-biotic stresses during this period (Palta et al., 1994; Barbottin et al., 2005).

**FIGURE 1:**
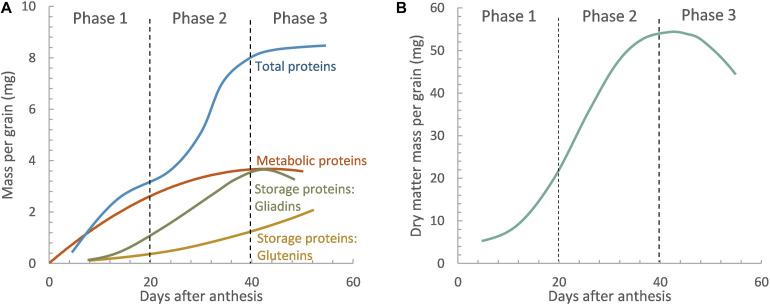
General scheme of protein fractions **(A)** and dry matter accumulation **(B)** in the wheat grain as a function of the days after anthesis (Phase 1: cell division, Phase 2: cell enlargement, and Phase 3: dehydration and grain maturation). Reproduced from Stone and Nicolas (1996) with permission from CSIRO Publishing.

Prolamins consisting of gliadins and glutenins account for 70–80%, while the non-prolamin part, including albumins and globulins, accounts for 20–30% (Tasleem-Tahir et al., 2012). Prolamins are the main storage proteins and condition the viscoelasticity of dough. In contrast, non-prolamins, called metabolic proteins, are important in cellular metabolism and contain more essential amino acids important for human health, such as aspartate, threonine, lysine, and tryptophan compared to prolamins (Tasleem-Tahir et al., 2012). Nonetheless, some studies have also reported high-molecular-weight albumins and certain globulins having a storage function (Gao et al., 2009; Dong et al., 2012). Albumins and globulins are accumulated first, during the first 10 days after the anthesis (Gupta et al., 1991; Stone and Nicolas, 1996). Although they accumulate through the whole grain-filling development stage, they represent a low percentage of the total protein amount at physiological maturity. This is because, after that, the accumulation of reserve proteins accounting for the highest percentage of the total amount at maturity starts (Stone and Savin, 1999; Triboi et al., 2003). There are suggestions in the literature about the influence of certain albumins on rheological properties of wheat flour dough, especially those associated with water absorption and resistance to extension (Osipova et al., 2012; Tomić et al., 2015). However, gluten proteins are primarily responsible for the viscoelastic properties of dough and ultimately the processing and end-use quality of wheat. Gluten is mainly composed of gliadins, soluble in alcohol, and glutenins, insoluble in alcohol–water solutions. Gliadins are the first reserve proteins deposited, around 5–10 days after fertilization, whereas glutenins are detectable 20 days after fecundation; both accumulate at the end of grain-filling (Panozzo et al., 2001; Figure 1). Gliadins provide the extensibility and viscosity of the dough, while glutenins contribute to elasticity and dough strength (Wieser, 2007). Equilibrium between gliadins and glutenins is advantageous to attain dough that does not need excessive mixing energy to reach peak development (Stone and Savin, 1999). Therefore, any factor affecting the length and rate of the grain-filling period may alter protein composition and reduce the GPC, thus modifying the dough properties (Jamieson et al., 2001).

The gluten constitution of a wheat-flour sample is set by the genotype, due to the configuration of the three high-molecular-weight glutenins (HMW-GS) subunits, the three low-molecular-weight glutenins subunits (LMW-GS), and six gliadin-coding loci (Wrigley et al., 2006). The gluten quantity is generally correlated with the GPC that is more influenced by growing conditions determined by temperature, rainfall, and soil fertility, as well as harvest, storage, and transport. Interactions between genotype and environment also have significant influence on wheat quality as some cultivars are more affected by growing conditions than others (Wrigley, 2009).

The interaction between different components of the wheat grain determines the processing and end-use quality. The growth of wheat grain is sigmoidal, beginning with a short lag phase when cells in the endosperm divide rapidly with little increase in dry weight, which determines the potential size of the grain (Brocklehurst, 1977; Hunt et al., 1991). During this lag phase, the sites where starch and proteins will accumulate are formed, and the first traces of starch and protein, mainly metabolics and gliadins, appear. This phase is followed by a longer and more rapid filling period when dry weight increases as a linear function until a maximum dry weight is attained (Brocklehurst, 1977; Jenner, 1991), at the end of which the rate of dry matter accumulation slows until physiological maturity when no further addition is made to grain weight (Figure 1).

Although considerable attempts have been made to further understand abiotic stresses, including N nutrition on milling, processing, and end-use quality of wheat (Triboi et al., 2000; Nuttall et al., 2017), there is less known about the effect of biotic factors, such as genotypic resistance to fungal diseases and their interaction with N fertilizers and fungicides on crop N dynamics, GPC, gluten content, milling, and dough properties, which have been only partly addressed in recent years.

Wheat diseases are responsible for 10–28% of yield losses worldwide (Bockus et al., 2001; Figueroa et al., 2018; Savary et al., 2019). Among them, foliar diseases have crucial importance. Foliar diseases caused by fungi-like rusts [stripe (yellow) rust *Puccinia striiformis* f. sp. *tritici* Westend., leaf rust *Puccinia triticina* Eriks], Septoria leaf blotch (*Zymoseptoria tritici* P. Crous), and powdery mildew (*Blumeria graminis* f. sp. *tritici* (DC. Speer) are ranked among the most important ones worldwide (Dean et al., 2012). Other important foliar wheat diseases are tan spot (*Pyrenophora tritici-repentis* (Died.) Drechs, anamorph *Drechslera tritici-repentis* (Died), spot blotch (*Cochliobolus sativus* S. Ito & Kurib., anamorph *Bipolaris sorokiniana* (Sacc.) Shoemaker), and Septoria nodorum blotch (*Phaeosphaeria nodorum* (Müller) Hedjar, anamorph *Parastagonospora nodorum* (Berk.) Quaedvl., Verkley & Crous).

Foliar diseases may influence the dynamics of carbohydrates and N, which determine grain yield and quality (Gaju et al., 2014). In addition, management practices such as N fertilization, genotypes, and fungicides may impact these effects in a differential way. Nitrogen fertilization may influence the severity caused by fungal diseases, generally increasing grain yield and modifying N dynamics and end-use quality (Castro et al., 2018; Schierenbeck et al., 2019a,b,c). Furthermore, fungicide applications can increase yield and cause a differential effect on the N remobilization, processing, and end-use quality parameters, depending on the nutritional habit of the pathogen (Fleitas et al., 2018a,b; Schierenbeck et al., 2019a,b,c). Moreover, the effects of fungicides on NREM and end-use quality can be different according to the type of fungicides used, due to their variable effects on leaf senescence and grain yield. Triazoles, which are characterized by a lively inhibitor of ergosterol, are one of the foremost groups of fungicides available to control foliar diseases in wheat. They are usually utilized in combination with strobilurins, which are synthetic derivatives produced by the Basidiomycete fungus *Strobilurus tenacellus* (Pers.), with a wide antifungal spectrum. Bayles (1999) mentioned that strobilurins could cause substantial yield increases, higher than those produced by conventional fungicides, because they have an ethylene-synthesis-inhibition property, which can cause a delay in leaf senescence. Furthermore, the incorporation of carboxamides (succinate dehydrogenase inhibitors) in triazole-strobilurin mixtures has resulted in better control of some foliar wheat diseases, such as tan spot and leaf rust (Fleitas et al., 2018a,b). On the other hand, the effects of fungal diseases and consequently of fungicides used to control them on GPC may vary according to the end-use quality of genotypes used (Puppala et al., 1998; Dimmock and Gooding, 2002c; Castro and Simón, 2016).

A review of the effects of fungal foliar diseases with different nutritional habits on crop dynamics, protein and gluten concentration, milling, and end-use quality in wheat is presented. The interaction among the diverse nutrition strategies of foliar pathogens with different N schemes, genotypes, and fungicide applications is also summarized. Controversial results, drawbacks, and gaps in research areas, and new insights and strategies to solve them and minimize the impact of those diseases on quality variables are discussed.

## The Interaction of Nitrogen Fertilization With Foliar Wheat Diseases Caused by Pathogens of Different Nutritional Strategies

Effects of foliar wheat diseases on yield and N dynamics and consequently on milling and end-use quality depends on several factors, including the severity of the diseases and the growth stage at which they occur. It has been reported that the nutritional habit of the pathogen and its interaction with N fertilization affect the severity of the diseases (Olesen et al., 2003) impacting N dynamics (Schierenbeck et al., 2019a,b,c).

**FIGURE 2:**
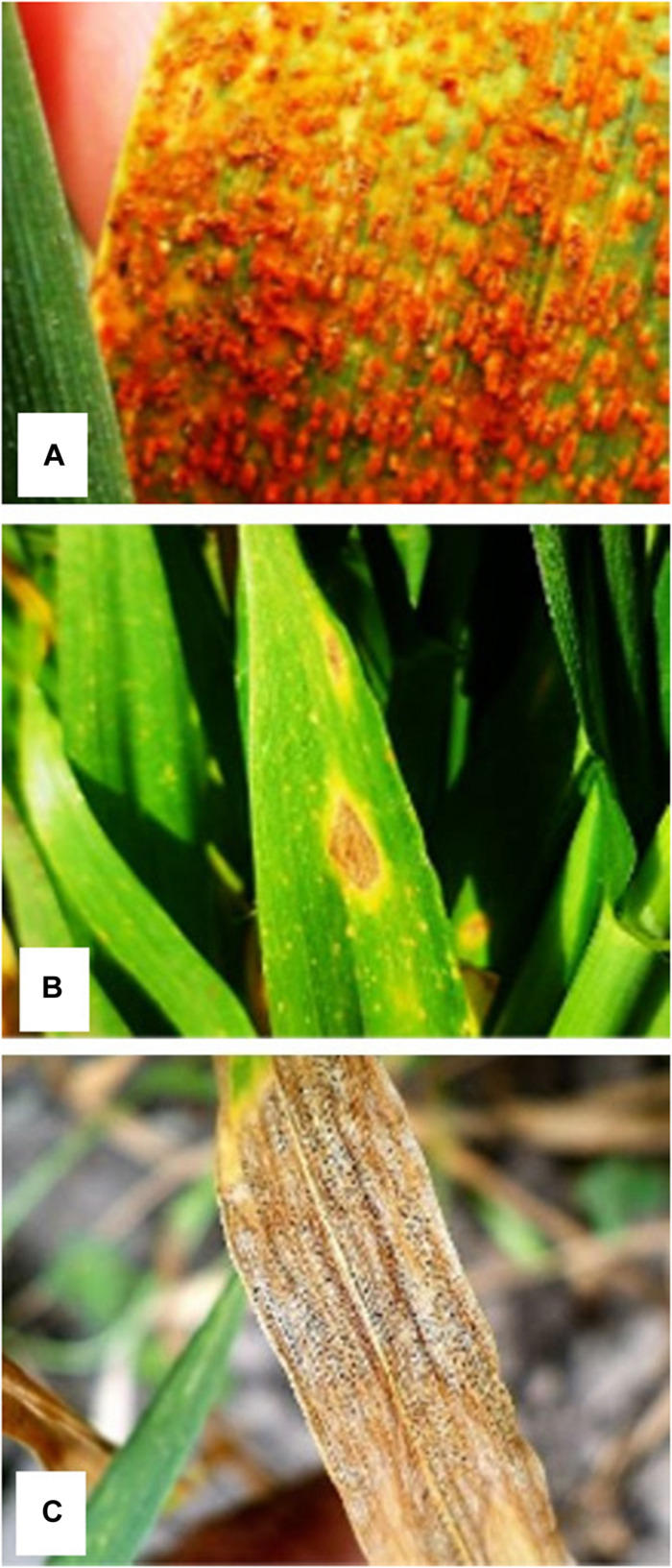
Disease symptoms caused by **(A)** *Puccinia triticina* (leaf rust), **(B)** *Pyrenophora tritici-repentis* (tan spot), and **(C)** *Zymoseptoria tritici* (Septoria leaf blotch).

According to their nutritional habit, pathogens can be classified as biotrophs, necrotrophs, or hemibiotrophs (Oliver and Ipcho, 2004). Necrotrophs (such as *P. tritici-repentis, Phaeosphaeria nodorum, Cochiobolus sativus*) are non-obligate parasites that kill host tissues as they colonize and grow on the contents of dead or dying cells (Stone, 2001). When a spore of *P. tritici-repentis* comes in contact with a leaf of a susceptible host, it germinates by forming a germ tube that penetrates the epidermal cells through an appressorium or stomates and forms a vesicle. Fungal growth proceeds intercellularly within the mesophyll layer. Three pathogenic toxins have been identified beyond the advancing hyphae within the infection process. Ptr ToxA induces the necrosis symptom. The other two toxins, Ptr ToxB and Ptr ToxC, induce chlorosis but on different host lines and cultivars (Wegulo, 2011). Conversely, biotrophs (such as *P. striiformis* f. sp *tritici, P. triticina, B. graminis* f. sp. *tritici*) are obligate parasites that acquire nutrients for growth and sporulation from living cells, and hence the pathogen must maintain host viability (Voegele and Mendgen, 2011) modifying source–sink ratio within the leaf, deriving host nutrients to the fungal mycelium (Scholes and Rolfe, 2009; Bancal et al., 2012; Ney et al., 2013), and secreting a limited amount of lytic enzymes (Cooper, 1984). The host response to biotrophs, which colonize the intercellular space using structures, namely haustoria, which takes in nutrients without disrupting the cell wall, tends to be more complex (Mendgen and Hahn, 2004). This response often results in rate depletion of photosynthesis, a loss of chlorophyll from the infected leaf, and an increase in the respiration rate (Scholes and Rolfe, 1995; Robert et al., 2005; Carretero et al., 2011). Furthermore, hemibiotrophs, such as *Z. tritici*, shift from an early biotrophic phase to a late necrotrophic phase. This term is applied to species that have an extended (4–14 days) asymptomatic phase taking nutrients from living cells at the beginning. For *Z. tritici*, two stages with five phases are documented (Ponomarenko et al., 2011). The whole asexual cycle lasts a minimum of 2–3 weeks without physically penetrating host cells, indicating an exchange of cell surface–localized molecules. The first phase occurred 0–24 h after contact: initial growth of the hyphae on the leaf surface. The second occurred 24–48 h after contact: host penetration via stomata. The third occurred 2–12 days after contact: intercellular biotrophic phase as hyphae extending within mesophyll tissue and obtaining nutrients from the plant apoplast (Ponomarenko et al., 2011). During this early phase of colonization, the fungus grows slowly, and it is very difficult to detect increases in fungal biomass (Keon et al., 2007). However, after that, for reasons that are unclear, wheat cells start to die, likely generated by the increased apoplastic nutrient availability due to the loss of the plasma membrane within the host. During this second stage, called necrotrophic growth, two phases were identified. The primary occurred 12–14 days after contact: a rapid change to necrotrophic growth consisting of the appearance of lesions on the leaf surface and collapse of the plant tissues. The second phase, which occurred 14–28 days after contact, is the further colonization of mesophyll tissue and formation of pycnidia with conidia in substomatal cavities of senescent tissue. Involvement of a toxin during the switch from biotrophic to necrotrophic growth is suspected but has not yet been proven (Ponomarenko et al., 2011). This classification of *Z. tritici* as a hemibiotroph has been discussed recently as detailed analyses of the asymptomatic phase show that the pathogen does not affect host growth, calling into question the biotrophic nature of this asymptomatic phase (Sánchez-Vallet et al., 2015). However, Precigout et al. (2020) also considered *Z. tritici* a hemibiotroph because it shows a long latent period, which is characteristic of other hemibiotrophs. It is possible to say that at least during the first phases of development, *Z. tritici* does not physically penetrate host cells as do necrotrophics, and the presence of toxins that can quickly induce necrosis in necrotrophic pathogens is not proved. Figure 2 shows the main foliar diseases representing biotrophic (*a, leaf rust), necrotrophic (b, tan spot), and hemibiotrophic (c, Septoria leaf blotch)* pathogens in this review.

In early N fertilization, the tillering increases, modifying the crop structure and affecting the disease severity (Savary et al., 1995). In addition, high N rates increase the green leaf area index (GLAI) and N concentration and prompt a delay in senescence due to a higher radiation interception and radiation use efficiency (RUE) (Walters and Bingham, 2007; Hawkesford, 2014). Moreover, Snoeijers et al. (2000) reported that N nutrition status of wheat plants could induce contrasting effects on the expression of different foliar diseases depending on the cultivar, environment, and type of pathogen. On the one hand, a high N supply may cause a greater susceptibility of wheat to fungal diseases, by creating a positive crop microclimate due to an enhancement on aboveground biomass (Neumann et al., 2004; Devadas et al., 2014) or increasing the N compounds necessary to pathogen growth (Hoffland et al., 2000). Conversely, N can also enhance plant defense (Solomon et al., 2003; Tavernier et al., 2007).

**FIGURE 3:**
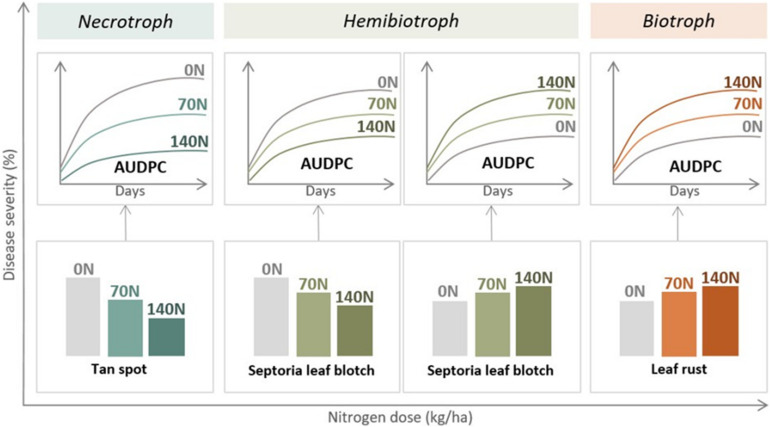
General diagram of the effect of nitrogen fertilization on three main foliar diseases of wheat in the same inoculated experiments. The nutritional habit of each pathogen causing disease is also indicated (AUDPC, area under disease progress curve; N, kg nitrogen/ha). Data indicated the most common response for biotroph and necrotroph pathogens and the different possible responses for hemibiotrophs.

The impact of a high N status of wheat plants on disease susceptibility was proved for biotrophic pathogens like *P. triticina* as they benefit from increased metabolite pools within the host cells (Jensen and Munk, 1997; Hoffland et al., 2000; Gerard et al., 2015; Fleitas et al., 2018a) increasing their severity under high N rates (Figure 3). This increased susceptibility at high N rates has been ascribed to the anatomical and biochemical modifications caused by N, together with the increase in organic N compounds, which are used as substrates by biotrophic pathogens (Dordas, 2008). In contrast, necrotrophic pathogens showed a more variable response to N (Hoffland et al., 2000; Long et al., 2000; Carignano et al., 2008; Fleitas et al., 2018a), probably because necrotrophs are ready to break down plant cell elements, allowing them to use a wider range of N sources (Solomon et al., 2003). Several researchers (Jones et al., 1990; Talbot et al., 1997; Hoffland et al., 1999; Snoeijers et al., 2000; Krupinsky et al., 2007; Carretero et al., 2010; Simón et al., 2011; Castro et al., 2018) have shown that tan spot severity (caused by a necrotrophic pathogen) decreased when N rates increased as low N availability results in weaker plants that are unable to defend themselves (Figure 3). For hemibiotrophic pathogens (such as *Z. tritici*) several investigations (Gheorghies, 1974; Prew et al., 1983; Broscious et al., 1985; Howard et al., 1994; Leitch and Jenkins, 1995; Simón et al., 2002, 2003) reported that N inputs increased the severity of the disease (behaving as a biotroph) (Figure 3). However, Johnston et al. (1979); Gooding and Davies (1992), and Fleitas et al. (2017) documented a decrease in the severity with increased N input with a differential response among genotypes, indicating a preponderance of the necrotrophic phase (Figure 3).

Controversial results, particularly for the specific case of a hemibiotrophic pathogen such as *Z. tritici*, can be mainly attributed to differences in total available N, different sources of N, or the possibility of an optimum N concentration for the development of this pathogen. The likelihood of an optimum N concentration in host plants for the development of *Z. tritici* was reported by Ishikawa et al. (2012). Furthermore, it should be considered that the hemibiotrophy of *Z. tritici* has been discussed (Sánchez-Vallet et al., 2015) due to the absence of alteration in the host growth during the asymptomatic phase. A question would be if under specific conditions some alterations in the host growth during that phase could emerge. These findings demonstrated that more research on the influence of N applications on the incidence of foliar diseases is needed. Joint experiments carried out recently with several pathogens separately inoculated in the same experiments already helped answer some questions (Fleitas et al., 2017, 2018a,b, Figure 3). Those experiments allowed us to determine that under the same environmental conditions and under artificial inoculations, *P. triticina* increased with N fertilization, whereas *P. tritici-repentis* and *Z. tritici* decreased, indicating behavior similar to a necrotrophic pathogen.

## Impact of Foliar Wheat Pathogens With Different Nutritional Habit on Crop Growth and Biomass Generation

Foliar diseases produced effects on the milling and end-use quality through modifications on the ratio N/carbohydrates in the grains. They may cause impacts on crop growth, reducing the number of grains and/or thousand kernel weights, impacting the N accumulation in the grains as a consequence.

In that way, the damage that foliar diseases produce depends not only on the severity of the pathogen but also on the incidence on the attributes responsible for the assimilation of carbon in the crop (Johnson, 1987; Waggoner and Berger, 1987). Foliar wheat diseases affect the GLAI and the healthy area duration (HAD) (Castro et al., 2018; Schierenbeck et al., 2019a,b,c) and consequently may have effects on dry matter losses, N accumulation in the grain, and GPC. More information is available on the effect of foliar diseases on dry matter yield losses (Bastiaans, 1993; Robert et al., 2004; Savary et al., 2006; Serrago et al., 2009; Carretero et al., 2010; Schierenbeck et al., 2016) than on N yield and GPC. As a general response, fungal foliar diseases decrease the crop growth rate (CGR) through reductions in HAD caused by foliar necrosis and accelerated death of tillers, reducing the ability of the crop to intercept and accumulate photosynthetically active radiation, and as a result, reducing the above-ground biomass accumulation and grain yield (Bancal et al., 2008; Serrago et al., 2009; Carretero et al., 2010; Ney et al., 2013). Wheat genotypes under standard practices in farmers’ fields (including N rates high enough to achieve yield goals and without fungicides) and under intensified practices (high N rates and fungicides) were recently compared. Results indicated that with a prevalent natural inoculum of *Z. tritici, P. tritici-repentis*, and *B. graminis* early in the season and *P. triticina* and *P. striiformis* late in the season, biomass response to intensified management was the driver for the yield increase, and that yield response was determined by the grain number but not by the grain weight (de Oliveira Silva et al., 2020). Within the same genotype, the biomass during the growing cycle, the rate of dry matter accumulation, and the grain-filling period duration may vary according to the date of sowing. Although no information is available, it is possible to speculate that likely within the same cultivars, a higher effect of pathogens could be evident in late sowing when phases are shorter.

**FIGURE 4:**
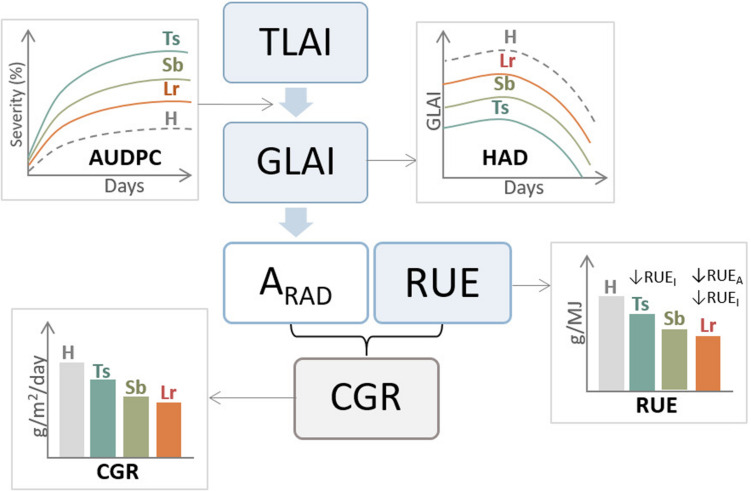
General diagram of the effect of three main foliar diseases of wheat, tan spot (Ts), Septoria leaf blotch (Sb), and leaf rust (Lr) on ecophysiological components of grain yield. AUDPC, area under disease progress curve; H, healthy (control treatment); TLAI, total leaf area index; GLAI, green leaf area index; HAD, healthy area index duration; A_*RAD*_, absorbed radiation; RUE, radiation use efficiency; CGR, crop growth rate; RUE_*I*_, Intercepted radiation use efficiency; RUE_*A*_, absorbed radiation use efficiency.

Inoculations of *P. tritici-repentis* generated more significant reductions in radiation absorption compared to *P. triticina.* By contrast, *P. triticina* reduced more RUE and CGR than *P. tritici-repentis*, implying that the photosynthetic system of the remaining healthy tissues infected by *P. triticina* is more negatively affected than under *P. tritici-repentis* infections (Schierenbeck et al., 2016). The negative effect of leaf rust on RUE and CGR could be associated with the nutritional habit of the fungus that generates major changes in the physiology of the host, reducing leaf N concentrations and enhancing assimilates consumed by leaf respiration. Increases in inoculum concentration decreased biomass generation, mostly conditioned by decreases in HAD and depletion on radiation absorption, with more significant reductions when the crop was inoculated with *P. tritici-repentis* than with *P. triticina* (Schierenbeck et al., 2016). Reductions in HAD were also found under *Z. tritici* inoculations (Castro and Simón, 2016; Figure 4).

Effects on the attributes related to the aboveground biomass production explain the yield losses that fungal leaf pathogens generate in wheat, leading to variations in the carbon/N balance in the grain that have an impact on flour quality. In this sense, yield can be expressed as the amount of biomass produced and the proportion derived to the reproductive organs (van der Werf, 1996). Another approach for yield determination considers the product of its numerical components, grain number/m^2^ and grain weight. Indeed, the yield is reduced predominantly through effects on the HAD and limitations on the number of grains per spike and grain weight (Cornish et al., 1990; Parker et al., 2004; Robert et al., 2004; Blandino et al., 2009; Serrago et al., 2011).

In wheat, the period between the beginning of the active growth of the spike and the brief period immediately after flowering, in which the stem and spike grow together, is crucial for yield determination because its main component, the grain number/m^2^, is being defined (Fischer, 1985). During this period, the crop is strongly limited by the photosynthetic capacity, so the maintenance of HAD is essential in order to maximize the CGR and provide assimilates to the spike (Miralles et al., 2000; Serrago et al., 2008). Taking this into account, the presence of foliar diseases during the critical period will cause (depending on GLAI reduction level) reduced grain yield due to its effect on different yield components (Yang and Zeng, 1989) and consequently, impacting milling and end-use quality. Depending on the growth stage when the fungal infection occurs, foliar diseases can affect the spikes/m^2^ (Leitch and Jenkins, 1995; Simón et al., 2002) generally associated with early epidemics of pathogens that survive on stubble and/or due to favorable conditions for the progress of these diseases during the early stages, mainly in susceptible cultivars. In addition, the grain number per spike (Madden and Nutter, 1995) and thousand kernel weights may be also commonly affected (Simón et al., 1996, 2011; Wang et al., 2004; Ishikawa et al., 2012; Castro and Simón, 2016), the latter associated with post-flowering infections. However, in some late infections, thousand kernel weight is not reduced for several reasons as compensation between thousand kernel weight and the number of grains when the latter is reduced, originating a higher accumulated absorbed radiation/number of grains ratio or due to compensation caused by other organs as spikes when the foliar area is affected by disease (Serrago et al., 2011; Carretero et al., 2011).

Furthermore, Rozo Ortega (2019) found that foliar diseases before flowering increased the source/sink ratio, whereas during the grain-filling period the source/sink ratio declined, consequently impacting the end-use quality. Moreover, it has been reported that foliar diseases also induce the crop to use the reserves of soluble carbohydrates stored in the stem to compensate the thousand kernel weight due to limitations in the source during the grain filling development stage (Bancal et al., 2007; Rozo Ortega, 2019).

## Physiological Effects of Fungicides to Control Foliar Diseases on Crop Growth

The impact of different fungicide molecules on physiological mechanisms involved in the green leaf area duration is a field of study of great interest that has received little attention in recent years. Different types of fungicides may exert a differential effect on crop growth and grain yield. In this sense, Wu and von Tiedemann (2001) reported that a mix of strobilurin and triazole molecules were able to delay canopy senescence due to an improved enzyme superoxide dismutase activity and high H_2_O_2_ levels that protected the plants from active oxygen species compared to untreated leaves. For their part, Ajigboye et al. (2014) documented that fungicides containing carboxamides and triazoles increased photosystem II efficiency in the flag leaf below, a response that showed a linear correlation with grain yield and biomass production even in the absence of disease. Positive effects of carboxamides and triazoles on photosynthetic activity due to HAD extensions and decreases in leaf temperature that led to a delay in leaf and ear senescence were also documented by Berdugo et al. (2012) under controlled conditions. Effects on photosynthesis efficiency, stomatal aperture, and plant transpiration were also reported following strobilurin applications in wheat (Kuznetsov et al., 2018). Another mechanism that would play a role in the senescence-delaying is the reduction in ethylene production reported by Gerhard et al. (1998) under strobilurin and triazole treatments. Furthermore, the chemical control of saprophytic fungi that are not able to attack plants but cause leaf senescence and grain yield reductions due to the energy cost of defense reactions were also reported as a beneficial effect of different types of fungicides (Bertelsen et al., 2001).

## Impact of the Nutritional Habit of Foliar Wheat Pathogens on Nitrogen Dynamics

The evaluation of the impact of foliar diseases on NREM and end-use quality faces several problems including the following: 1-Assesments under natural infections implies that the individual consequence of each pathogen is not easily determined. 2-The use of artificial inoculations allows us to determine the effect of the prevalent inoculated pathogen, which usually competes with other pathogens due to natural infections, but the latter is not completely eliminated. Several experiments have been carried out under natural infections and a few under artificial inoculations with specific pathogens. Nitrogen inputs may also interact with diseases and the fungicides used to control them, causing differential effects on N dynamics and consequently on GPC and gluten concentration.

Accumulation and N redistribution are important processes determining yield and grain quality (Simpson et al., 1983; Gaju et al., 2011). As mentioned previously, N in grains mainly comes from N accumulated before anthesis, and the amount of NREM is dependent on the N accumulated in anthesis (Gaju et al., 2014). Therefore, an early high N availability or late N supply can increase N in grains and, consequently, GPC.

In addition, several researchers have mentioned that NREM is involved in the control of leaf senescence (Masclaux et al., 2001; Uauy et al., 2006). At the beginning of grain development, N accumulation is source and sink regulated; however, during the grain filling, N accumulation was always limited by the source supply from vegetative tissues, even when soil N was non-limiting (Martre et al., 2003). The increase in N remobilization efficiency to slow down senescence may be critical for maintaining a longer photosynthesis period during grain-filling to achieve higher yields, but in bread-making cultivars this may not be beneficial due to the negative impact on N in the grain and consequently in GPC. The onset of post-anthesis senescence was negatively correlated to NREM under low N availability, but not under high N supplies (Gaju et al., 2014). Furthermore, NREM variations in N yield depend not only on NREM but also on both NPA and biotic and abiotic stresses during the grain-filling period (Barbottin et al., 2005).

Earlier reports about the effect of pathogens with differential nutritional strategies on the host have mainly been carried out under natural infections of a complex of pathogens or addressing the effect of single pathogens (Gooding et al., 2005; Bancal et al., 2008; Devadas et al., 2014). Hence, the individual effects of necrotrophic, biotrophic or biotrophic pathogens on crop N dynamics and grain quality traits are not easy to discriminate. However, it has been reported that when biotrophic pathogens affect wheat plants, the infection can be more disturbing to N deposition and partitioning to the grain than to dry matter partitioning and deposition (Dimmock and Gooding, 2002c). On the other hand, necrotrophic and hemibiotrophic pathogens have been reported to affect primarily the carbohydrates accumulation (Puppala et al., 1998; Gooding et al., 2007), while fungicides reverse this response (Fleitas et al., 2017), causing an increase in GPC.

The severity caused by the biotrophic pathogen *P. triticina* has greater effects on GPC than in dry matter partitioning and deposition in the grain, increasing protein concentration in leaves and stems and reducing GPC (Caldwell et al., 1934; Greaney et al., 1941). Although biotrophic pathogens also affect most N accumulation in the grain, they also reduce GLAI, radiation interception, and RUE, and cause premature senescence, reducing photosynthesis and translocation (Bryson et al., 1995; Lucas, 1998; Schierenbeck et al., 2016). Sugar and amino acids retention in diseased leaves and pustules of *P. triticina* leading to restrictions on the normal remobilization of assimilates to developing grains and decreases on N harvest index and N remobilization efficiency have also been reported (Walters, 1989; Dimmock and Gooding, 2002c; Schierenbeck et al., 2019a,c). Furthermore, Debaeke et al. (1996) determined that leaf rust, water shortage, and high temperatures affected dry matter deposition during grain-filling, generating a high GNC, indicating that environmental factors can also influence the differential effects of pathogens with distinctive nutritional habits. They also found that GNC was related to N absorption when N availability was the main restricting factor and to the N harvest index when drought or foliar diseases limited wheat yield.

Furthermore, it is also important to consider the time of infection. On the one hand, Bastiaans (1993) determined that when epidemics of foliar diseases occur before flowering in cereals, they reduce the absorption of N, but rarely affect the NPA. On the other hand, when infections occur after flowering, Barbottin et al. (2005) found that the impact of the environmental factors on the association between N uptake at flowering and NREM varied according to NPA, genotype, and disease pressure. They also reported that disease-resistant genotypes keep N remobilization efficiency more stable under high disease pressure. Moreover, Bancal et al. (2008) determined that although NPA accounted for a third of N yield and NREM for two thirds in healthy and diseased crops affected by late epidemics, variations in N yield were more correlated with NPA than with NREM, and they suggested that the latter is not associated with N yield in healthy crops. In addition, extending HAD through adequate climatic conditions, genetic resistance, or fungicide applications can be directly related to increases in N stored in grains (Gooding et al., 2005).

**FIGURE 5:**
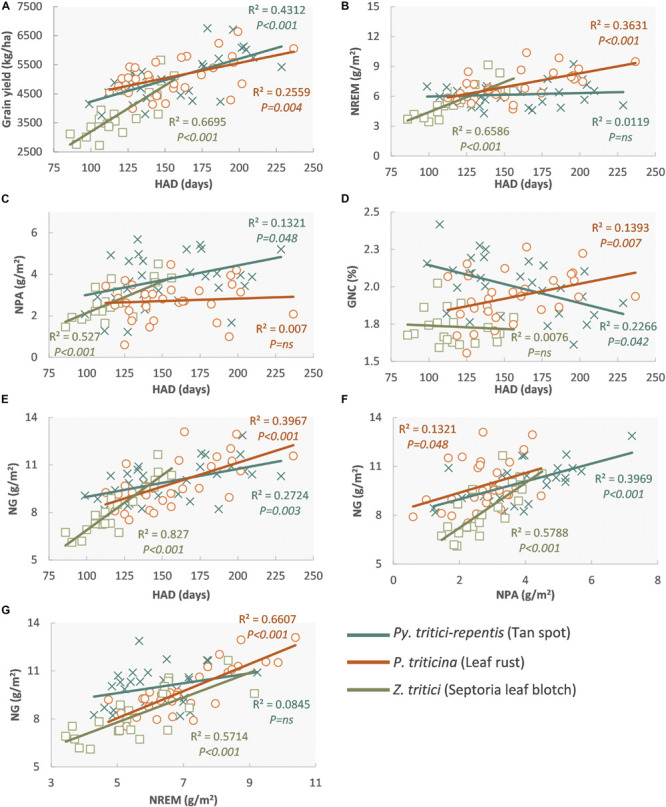
Regression between healthy area duration (HAD) and grain yield **(A)**, N remobilization (NREM) **(B)**, N post-anthesis uptake (NPA) **(C)**, grain N concentration (GNC) **(D)**, and N accumulated in grains (NG) **(E)**; N accumulated in grains (NG) and NPA **(F)**, NREM **(G)** in wheat inoculated with *P. tritici-repentis* (**×**), *Z. tritici* (□), and *P. triticina* (○). Each data point represents the means of each cultivar for three replications, regression fitted to data for each pathogen and three levels of inoculations. Reproduced from Schierenbeck et al. (2019a) with permission from Elsevier.

Under separated artificial inoculations with a necrotrophic (*P. tritici-repentis*) and a biotrophic (*P. triticina*) pathogen in the same experiment, NREM was more reduced by leaf rust infections through reductions in HAD and CGR compared to tan spot. Furthermore, *P. triticina* reduced N remobilization efficiency, GNC, and N stored in grains, whereas *P. tritici-repentis* increased GNC (Schierenbeck et al., 2019a,c; Figure 5). This higher impact of *P. triticina* on N dynamics, caused by the retention of N in green tissues, pustules, and mycelium of the pathogen that act as a secondary sink, has been found in other biotrophs and hemibiotrophs (Walters, 1989; Gooding et al., 2005; Ney et al., 2013). In inoculated experiments, *Z. tritici* also reduced NREM significantly, whereas NPA was less affected, producing lower GNC (Castro et al., 2015; Figure 5). Inconveniences in the nutrient balance of the crop reducing photosynthesis and remobilization of assimilates also have been reported as one of the main consequences of leaf rust (Lucas, 1998).

Negative effects exerted by foliar fungal pathogens on crop N dynamics could be reverted by management practices such as N fertilization, fungicide applications, and genetic resistance. Fungicide applications, mainly including specific mixtures of triazoles and strobilurins, extended the HAD and the duration of N filling in the grain, improving variables of N dynamics (Ruske et al., 2003; Barbottin et al., 2005; Gooding et al., 2005; Bancal et al., 2008; Ishikawa et al., 2012). Recently, Schierenbeck et al. (2019c; 2019b) documented that interactions between N applications and fungicide mixtures, including triazole + strobilurin and triazole + strobilurin + carboxamide, can reverse the detrimental impact caused by *P. tritici-repentis* or *P. triticina* infections on NREM, NPA, GNC, and N stored in grains. The triple fungicide mixtures produced greater increases in HAD and area under disease progress curve reductions, increases in grain yield, NREM, NPA, and N stored in grains with respect to triazole + strobilurin fungicides under high N rates and *P. tritici-repentis* infections (Schierenbeck et al., 2019b). Similarly, combinations of triple fungicide mixtures and high N rates surpassed double mixtures at high N rates in the effects on NREM, NPA, N stored in grains, GNC, and grain yield and caused significant increases on N remobilization efficiency and N harvest index under *P. triticina* inoculations (Schierenbeck et al., 2019c). These results showed that the use of new fungicidal molecules in combination with N fertilization could be a key tool to increase the efficiency of N utilization in wheat, reduce the incidence of disease, and improve grain quality parameters with differential responses, depending on the nutritional strategies of the main pathogen present.

A broad genotypic variation on NREM has been reported by Cox et al. (1985a; 1985b), Gooding et al. (2005), Kichey et al. (2007) and also by Barbottin et al. (2005) evaluating NREM against infections of *P. triticina* and *P. striiformis*, while Castro et al. (2015) did not find differences in the NREM in genotypes inoculated with *Z. tritici* as it may depend on the genotypes used. Additionally, variations in NPA among genotypes were reported (Cox et al., 1985a,b; Kichey et al., 2007; Bahrani et al., 2011). For instance, Schierenbeck et al. (2019a) found genotypic variations in NREM, NPA, N stored in grains, and GNC under separate inoculations with *P. triticina* and *P. tritici-repentis.*

Regarding the effect of combinations of fungicide applications and N doses in different genotypes, Mascagni et al. (1997), Ruske et al. (2003); Brinkman et al. (2014), and Schierenbeck et al. (2019c; 2019b) reported that the highest responses in crop N dynamics were detected in genotypes with greater susceptibility to diseases, allowing better discrimination of the effects of pathogens with different nutritional strategies under increases in N fertilization. In the same way, the application of foliar fungicides has shown variable responses for yield (Roth and Marshall, 1987), but in general, it has been beneficial when it was used for cultivars susceptible or intolerant to diseases and with high yield potential (Kelley, 2001; Olesen et al., 2003).

**FIGURE 6:**
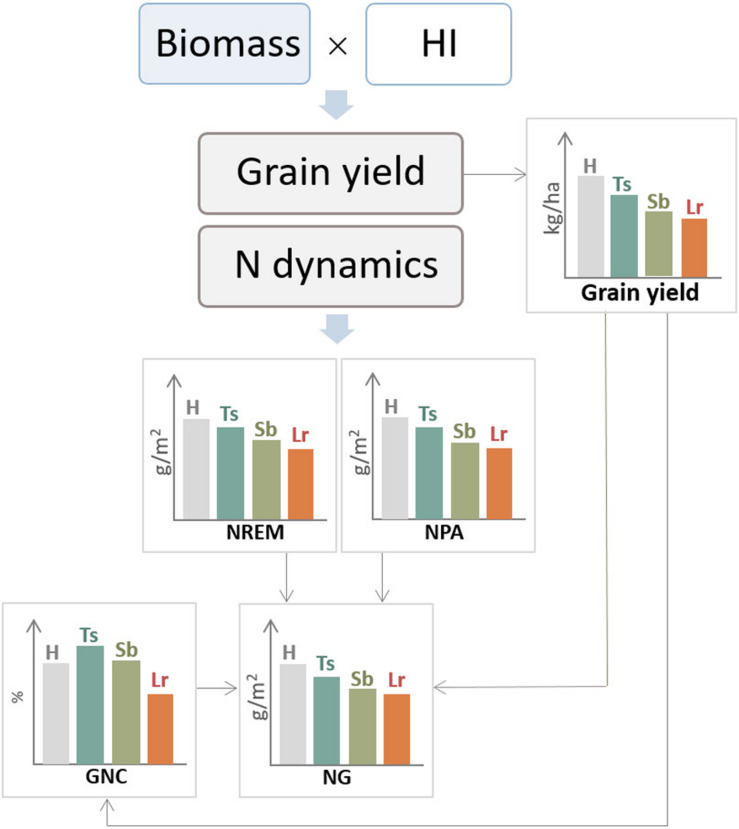
General diagram of the effect of three main foliar diseases of wheat, tan spot (Ts), Septoria leaf blotch (Sb), and leaf rust (Lr), on ecophysiological components of grain yield and N dynamics. H, healthy (control treatment); NG, N accumulated in grains; NREM, nitrogen remobilization; NPA, N post-anthesis absorption; GNC, grain N concentration.

As mentioned previously, necrotrophic pathogens such as *P. tritici-repentis* reduces HAD and the absorbed radiation, which decreases the CGR (Serrago et al., 2009; Schierenbeck et al., 2016). If these effects occur during the grain-filling stage, given that almost 65–80% of the N was already accumulated at anthesis, the GPC may increase due to a concentration effect. Conversely, biotrophic pathogens such as *P. triticina*, in addition to reducing the absorbed radiation (Bryson et al., 1995), affect the RUE (Schierenbeck et al., 2016), which limit their remobilization to grains and N harvest index, generally causing a reduction in GNC (Dimmock and Gooding, 2002c; Schierenbeck et al., 2019a; Figure 6).

Results showed that pathogens with different nutritional habits impact N dynamics differentially. Particularly in recent years, important contributions have been made with pathogens inoculated separately in the same experiments, allowing better discrimination of individual microorganisms. New fungicide molecules in combination with N applications have reduced the effect of biotrophic pathogens on the N dynamics. These data deserve to be incorporated in simulation models, increasing possibilities for the management of those diseases.

## The Effect of Foliar Wheat Diseases on Milling, Processing, and End-Use Quality

### Effects on the Balance Between N and Carbohydrate Accumulation

The balance between N and carbohydrate accumulation in the grain may produce variation in thousand kernel weight, affecting milling and GPC. Both components are accumulated independently, starting with metabolic proteins and gliadins and then glutenins and starch deposition. The increase in the grain mass, mainly due to a high carbohydrate accumulation, may cause a dilution in GPC, whereas the reduction in thousand kernel weight, in addition to producing shriveled grains, may concentrate GPC. As mentioned previously, the N accumulated in the grains mainly comes from the N deposition in vegetative tissues before anthesis and is remobilized to the grains during the grain-filling period (Palta and Fillery, 1995). The accumulation of carbohydrates depends mainly on the current photosynthesis during grain development (Blum, 1998). The effect of foliar diseases on the N/carbohydrates ratio in the grains depends on the magnitude of the reduction they generate on each component in the vegetative organs, the nutritional habit of the pathogens being a key factor. It also depends on the ability of the crop to compensate shortfalls of carbon generated from current photosynthesis by translocation of carbohydrates to the filling grains (Robert et al., 2002).

Abiotic and biotic stresses during the grain filling inhibit current photosynthesis, generating a reduction in post-anthesis carbohydrates assimilation but increases in the remobilization of reserves. For that reason, the relative contribution of stem reserves as soluble carbohydrates to the grain mass fluctuates according to the growing conditions, biotic stresses, and genotypes (Palta et al., 1994; Blum, 1998). Furthermore, when grain filling depends on those reserves, the rate of remobilization and the duration of grain development are very important to determine the grain weight (Blum, 1998). Some tolerant wheat genotypes did not reduce the thousand kernel weight under epidemics of *Z. tritici*, which can be attributed to a compensation through carbohydrates supply from healthy tissues to the grains (Zilberstein et al., 1985).

Early sowing dates where *Z. tritici* was the predominant pathogen caused a low reduction in soluble carbohydrates, whereas late sowing dates where *P. graminis* was the main pathogen implied important reductions in soluble carbohydrates likely attributed to the strong demand due to the grain filling. Biotrophic pathogens caused reductions in photosynthesis as necrotrophics but also a direct consumption of photoassimilates, causing higher reductions in soluble carbohydrates (Serrago et al., 2011). Additionally, differences in the growth duration of the whole cycle and the duration of the grain-filling period can cause differential effects on the accumulation of soluble carbohydrates determining different concentrations in the grain. The date of sowing of the cultivars may also cause variation in the accumulation of carbohydrates, causing different N/carbohydrates balances in the grain, although no information is available using the same genotypes.

Managing foliar diseases helps grain-filling, reducing the phenomenon of grain shriveling, which is related to low flour extraction rates in milling. In general, fungicides applied at flag leaf stage have the greatest effect on carbohydrates accumulation, yield, thousand kernel weight, and test weights when severe diseases are controlled. For instance, MacLean et al. (2018) noted that fungicide application at anthesis to control leaf spotting diseases resulted in yield benefits, and improved grain quality measured as test weight and thousand kernel weights; however, it may dilute GPC. Ultimately, foliar diseases might also affect processing and end-use quality of wheat as the behavior of the dough is associated with the type and amount of protein present in the flour (Simón et al., 2013). Strong evidence suggests that the effect of disease control on milling, processing, and end-use quality interact with the genotype, climate and agronomic practices, pathogen type, time and intensity of infection, among other factors discussed here. Several investigations of these variables have been conducted under natural infections caused by fungi, where, in some cases, prevalent pathogens in the field are not specified. Only a few investigations have been carried out under artificial inoculations with specific pathogens.

### The Effect of Foliar Diseases and Their Control of Milling Quality and Alpha-Amylase Activity of Wheat

Milling quality of bread wheat is referred to as the aptitude of a cultivar to produce high levels of flour (Guzmán et al., 2016). As mentioned previously, the most important traits associated with milling quality are the grain morphology, thousand kernel weight, grain hardness, and grain density (test weight). Millers prefer sound, large grains, well filled and without shriveling, with absence of sprouted grain (Carson and Edwards, 2009).

Test weight is the weight of 100 liters of wheat evaluated with the Schopper chondrometer. It is a measure of the density and compactness of the grain (Carson and Edwards, 2009; Edwards, 2010). With larger grains, the endosperm/bran ratio is greater, and therefore the potential yield of flour as well. On the other hand, grain hardness refers to the texture of the wheat grain endosperm. Hard grains require more power consumption in the mill to fracture and mill a selected weight of the sample. Additionally, the harder the grain, the more damaged the starch granules in the flour, which is a source of fermentable sugar during baking but also will determine the water absorption of the flour. Thus, grain hardness significantly affects milling along with processing and end-use quality (Morris, 2002; Pasha et al., 2010).

Foliar diseases affect milling quality through less dry matter accumulation in the mature grain that results from decreases in the photosynthetic area of leaves, through reductions of the radiation intercepted and RUE, but also increases in the respiration rate, interfering with photosynthates translocation (Figure 4). Herrman et al. (1996) documented premature senescence of the flag leaf in rusted plants, which shortened the grain-filling and restricted N remobilization to the grains.

Regardless of the pathogen nutritional habit, diseased plants may generate fewer tillers and set fewer and smaller grains per spike, usually shriveled and with lower milling quality than healthy plants. Shriveled grains occur because the time available for the grain to fill is shortened (i.e., the dry matter assigned to the grain is reduced) but also because the disease leads to earlier maturity of the plant (Agrios, 2005). Shriveled grains have low test weights, contribute to impurities in the flour, have lower flour yield, higher ash content, lower metabolizable energy content (Gaines et al., 1997; Gooding and Davies, 1997), and increased hardness (Buendía-Ayala et al., 2019) compared to well-filled kernels. They may also have inferior baking qualities (decreased cookie diameter) despite their greater grain and flour protein content compared to well-filled grains (Gaines et al., 1997).

The starch composition (amylose/amylopectin ratio) may have an influence in milling properties as well as rheological dough characteristics and may be affected by foliar diseases. The amylose/amylopectin ratio has been found negatively correlated with vitreous kernels, wet gluten, and flour protein content in some experiments and positively correlated with flour yield, test weight, and (sodium dodecyl sulfate) sedimentation test or farinograph water absorption in some others. In a combination of three environments, starch content played a more important role in quality than amylose/amylopectin ratio. Starch content was positively correlated with single kernel diameter, test weight, and thousand kernel weight and negatively correlated with loaf volume, alveograph strength, wet gluten content, flour protein, and farinograph absorption (Labuschagne et al., 2007), due to a reduction in the N/carbohydrates ratio. Genotypes and environment affected the starch content, and a high content sometimes caused lower bread-making quality. Although the information is scarce, it has been found that some foliar pathogens, such as *B.graminis*, caused a decrease in amylopectin content (Li et al., 2018) and a decrease in the ratio of amylose/amylopectin and grain fullness (Morris and Rose, 1996).

Fungicide applications significantly increased both grain yield and milling quality, on the order of 10–32% compared to the control without fungicide according to the type of active ingredient, the growth stage of the application, genotype, and disease controlled (Castellarín et al., 2004). In general, when the severity of disease is significant, effects on test weight and/or thousand kernel weight are coincident. Varga et al. (2007) found that the test weight increased after triazole application, mainly for disease-susceptible genotypes at high N rates, although no information about the prevalent diseases is indicated. When predominant diseases were recorded, losses in thousand kernel weight and test weight, reducing milling yield under *P. triticina* and *P. graminis* natural infections (Keed and White, 1971) or *P. striiformis* infections were found (O’Brien et al., 1990). Several experiments under triazole applications reported that thousand kernel weight and test weight were improved in crops affected by *P. tritici-repentis* and *Z. tritici* (MacLean et al., 2018) or *Z. tritici* (Ruske et al., 2003) or *Z. tritici, P. tritici-repentis* and *P. triticina* infections (Puppala et al., 1998). More recently, long-term field experiments under natural infections with a predominance of *Z. tritici, P. striiformis*, and *B. graminis* found that different carboxamides, strobilurins, and sterol-demethylation inhibitors increased thousand kernel weight, test weight, and grain starch content (Matzen et al., 2019).

An additional important test in the milling industry to determine the acceptability of a wheat grain lot is the Hagberg falling number (HFN) analysis, which quantifies the level of alpha-amylase activity in the grain. Under rainy conditions before harvest, wheat seeds may germinate within the spike, and associated enzymes activate. This phenomenon, known as “pre-harvest sprouting” (Carson and Edwards, 2009), causes an increase in alpha-amylase activity that is undesirable for bread production. The HFN can be measured through a rapid test, by timing the descent of a stirrer through a hot suspension of ground wheat in water (Hagberg, 1961). The greater the amount of alpha-amylase, the faster the stirrer will fall, determining low values of HFN. An additional cause for high alpha-amylase activity is late maturity alpha-amylase, which is a genetic defect existing in some genotypes related to rapid temperature change after flowering (Newberry et al., 2018).

It has been suggested that the alpha-amylase level is affected by agronomic practices, such as fungicide applications to control foliar diseases (Gooding et al., 1987; Kettlewell, 1999; Matzen et al., 2019). Extending HAD in wheat with fungicides may increase alpha-amylase activity and/or reduce HFN associated with increased grain size and weight (Gooding, 2017). The mechanism by which fungicide affects alpha-amylase activity, and consequently HFN, has yet to be clearly defined, but in the absence of sprouting, alpha-amylase activity seems to be increased by slow grain drying (Gale et al., 1983; Kettlewell, 1997). Fungicide interactions that increase predisposition to late maturity alpha-amylase may also cause a hormonal effect. Numerous studies have reported HFN reductions after triazole and/or strobilurin applications under the prevalence of several pathogens (Ruske et al., 2003, 2004; Wang et al., 2004); however, values often remain above the 250 seconds, which are required standards for bread-making (Draper and Stewart, 1980). Dimmock and Gooding (2002a) found that although HFN was reduced by fungicide applications, this effect depended on the cultivar, fungicide type, and the prevalent pathogen, as controlling severe rust did not affect HFN. More recently, Gooding (2017) stated that the effect of fungicides on HFN does not seem to depend on the type of pathogen controlled or the mode of action of the protectant. One possible explanation is that the effect of fungicides may depend on how much the thousand kernel weight increase causes a dilution in the alpha-amylase activity. Clearly, the information available is scarce, and further investigations to elucidate how fungal diseases impact HFN, particularly with different genotypes and N fertilization rates, are necessary.

### The Effect of Foliar Diseases and Their Control on Processing and End-Use Quality of Wheat

#### Main Variables of Processing and End-Use Quality Affected by Foliar Diseases

Foliar diseases may affect grain protein and gluten concentration, with consequences on processing and end-use quality variables. Among those variables, the alveograph estimates the gluten strength of dough by means of the Chopin^®^ Alveograph measuring (i) dough tenacity (AlvP), which is the force necessary to blow a bubble of dough; (ii) dough extensibility (AlvL), which is the extensibility of the dough before the bubble breaks; (iii) the AlvP/L ratio, which is the relationship between dough tenacity and dough extensibility; and (iv) dough strength (AlvW), which is the area under the curve and is proportional to the energy required to cause the dough bubble to break. A farinograph determines the resistance of dough to mixing. It is measured by the Brabender^®^ Farinograph, evaluating (i) flour water absorption (FarA), which is the amount of water necessary to center the farinograph curve on the 500-Brabender unit (BU) line and is related to the amount of water required for a flour to be optimally processed into end products; (ii) dough development time (FarB) (peak time), which is the time from water added to the flour until the dough maximum consistency, providing an indication of optimum mixing time under standardized conditions; (iii) dough stability time (Far D), which is the time difference between the point where the top of the curve first intercepts the 500 BU line and the point where it leaves the 500 BU line. Dough stability time indicates the time the dough maintains the maximum consistency and is a good indication of dough strength. Finally, (iv) dough softening degree or departure time (FarE) is the time when the top of the curve leaves the 500 BU line, indicating the time when the dough is beginning to break down and dough consistency starts falling below optimum during kneading.

Another end-use quality test is Zeleny sedimentation, which is based on the ability of gluten-forming proteins to soak in water, indicating the gluten strength and baking potential. The mixing tolerance index is the difference in BU between the top of the curve at peak time (FarB) and the top of the curve 5 min later, indicating the degree of softening during mixing. The extensograph determines the resistance and extensibility of the dough by evaluating the force required to stretch the dough with a hook until it breaks. The resistance to extension is a measure of dough strength and the extensibility determines the amount of elasticity in the dough and its ability to stretch without breaking. Finally, the loaf volume gives end users information on flour quality traits. It is determined by measuring the volume of bread obtained after the bread-making process by means of a rapeseed displacement using a volume-meter.

#### Effect of Foliar Diseases Caused by Biotrophs on Processing and End-Use Quality of Wheat

**FIGURE 7:**
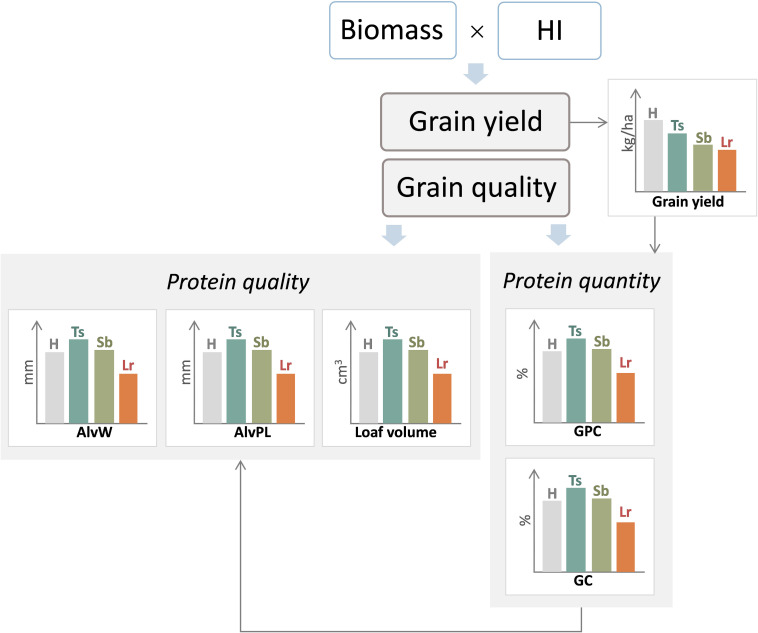
General diagram of the effect of three main foliar diseases of wheat, tan spot (Ts), Septoria leaf blotch (Sb), and leaf rust (Lr), on grain yield and bread-making quality. H, healthy (control treatment); AlvW, dough strength; AlvP/L, dough tenacity/dough extensibility ratio; GPC, grain protein concentration; GC, gluten concentration; HI, harvest index.

As mentioned previously, biotrophs can produce greater effects on N accumulation in the grain than on carbohydrates (Caldwell et al., 1934; Keed and White, 1971; Park et al., 1988; Herrman et al., 1996; Dimmock and Gooding, 2002c; Rozo Ortega, 2019), and thus, GPC increases by fungicide applications (Figure 7). More recently, artificial-inoculated experiments also showed that the control of leaf rust increased grain protein and gluten concentration, even when thousand kernel weight increased, indicating that there was no evidence of protein dilution (Fleitas et al., 2015, 2018b; Figure 7).

Although the tendency of the impact of biotrophic pathogens is clearly toward a decrease in GPC as N is generally more affected than assimilated carbohydrates, some controversial results have been reported. O’Brien et al. (1990) documented that when *P. striiformis* was predominant, GPC increased due to the concentration effect generated by shriveled grains and Matzen et al. (2019) under natural infections generated by *Z. tritici, P. striiformis*, and *B. graminis* found that disease control reduced GPC, also due to a dilution effect. Furthermore, different responses depending on the genotype have been found (Everts et al., 2001; Buendía-Ayala et al., 2019), sometimes varying with the resistance level (Conner et al., 2003).

Results indicate that in some experiments a high reduction in thousand kernel weight due to biotrophic pathogens could be decisive for causing a concentration in GPC. In other cases, several diseases are present together, causing inconsistent effects, mainly under natural infections. The end-use quality of genotypes can also play a role, as those with higher bread-making aptitude tend to maintain higher GPC values. In addition, N availability may influence the results as a high N availability can increase the incidence of some biotrophs, such as rusts, but also tends to increase GPC. Furthermore, Robert et al. (2004) observed an N threshold from which the N fertilization impact is more important on GPC than on leaf rust severity. In addition, in “modern” cultivars, mainly affected by *P. triticina* but also by *P. tritici-repentis*, the reduction in protein and gluten concentration was higher than in “old” cultivars due to a greater number of grains determining less N available for each grain, causing a dilution effect (Rozo Ortega, 2019). Under different stresses, the remobilization of reserves from the stem to the grains can increase, although some contradictory effects have been reported in genotypes affected by *P. graminis* and *P. striiformis* (Chang et al., 2013) due to the susceptibility of the genotypes. In that way, biotrophic pathogens may affect translocation of N into the grains but also the accumulation of soluble carbohydrates due to reductions in the HAD and the increase in senescence (Dimmock and Gooding, 2002c; Matzen et al., 2019). Therefore, the effect on the GPC may depend on the magnitude to which N and soluble carbohydrates are affected.

Recently Fleitas et al. (2018b), in inoculated experiments with *P. triticina*, reported that rusted plants had shorter grain-filling periods and reduced gluten concentration with respect to protected plots with different fungicide mixtures (Figure 7). Even though gluten concentration followed the same tendency as GPC, the effects of leaf rust in the untreated plots compared to healthy plants were more important on gluten concentration than on GPC, particularly under higher N status, indicating that the disease may alter the GPC composition, likely reducing the proportion of insoluble protein fractions.

On the other hand, the effect of biotrophic pathogens in dough rheology is not as consistent or as marked as that observed on GPC, and the magnitude and direction of those effects have been understood less clearly. It has also been reported that under *P. triticina* artificial inoculations, the alveogram parameter AlvP was more decreased than AlvL, resulting in a minor AlvP/L ratio (Fleitas et al., 2018b; Rozo Ortega, 2019), and loaf volume was also affected (Fleitas et al., 2018b). O’Brien et al. (1990) reported that under *P. striiformis* epidemics, the dough development time from the farinograph (FarB) was shorter and mixing tolerance and extensograph maximum resistance were lower for susceptible varieties, whereas the loaf volume was not affected. Conversely, Feng et al. (2014) addressed the influence of powdery mildew infection and found that rheological variables of the dough, such as extensograph and farinograph parameters, increased in diseased plants, whereas Buendía-Ayala et al. (2019) found that the dough kneading time following fungicide applications to control yellow rust decreased.

Interactions between different types of fungicides and N fertilization for gluten concentration, rheological properties, and loaf volume were also found in experiments inoculated with *P. triticina*. Fungicide treatments reduced wet gluten content at low N rates, but this effect was reverted at the highest N dose, where the treatment with triazole + strobilurin + carboxamides evidenced higher values compared to the control and the triazole + strobilurin treatment (Fleitas et al., 2018b). Furthermore, AlvP and AlvW augmented more under N applications when experiments were treated with the triple fungicide mixture compared to the double mixture and the untreated plots. Values of AlvL increased independently of the fungicide type and N rates. Additionally, farinograph parameters FarA and FarB increased under the triple fungicide mixture and FarE under all fungicide treatments at rising N rates. Moreover, fungicide applications generated greater increments of loaf volume with increasing N rates (independently of the fungicide type) than the untreated plots (Fleitas et al., 2018b). Blandino and Reyneri (2009) reported that AlvW increased under N application treatments following a triazole-only and a triazole + strobilurin fungicide application and a tendency to increase the AlvP/L under fungicide treatments compared to the unprotected treatments.

The literature available on the effects of the diseases on rheological parameters is characterized by many inconsistencies, and more research is necessary. For instance, many of these studies have a limited number of genotypes, and hence the lack of distinction in the response of the genotypes according to their bread-making aptitude could have led to the inconsistent results (Dimmock and Gooding, 2002c). In addition, gluten composition may influence these discrepancies as the effect of foliar diseases on gliadins/glutenins ratio has not been addressed. Gliadins fraction, which tends to accumulate earlier in the grain, affect the AlvL values, whereas glutenins (which accumulate later) affect AlvP values; thus the effect of diseases could be different, depending on which phase of grain development is more affected. This fact has been also confirmed under inoculations with *P. triticina* as the decrease in AlvP/L and consequent reduction in loaf volume compared to the protected treatments could be attributed to changes in gluten composition (Fleitas et al., 2018b). Furthermore, the ratio between gluten fractions with different molecular weight can be modified. In that sense the leaf rust decreased high-molecular-weight glutenins, increasing dough extensibility (Fullington and Nityagopal, 1986).

Knowledge is even more limited regarding the effect of foliar diseases on albumin and globulin accumulation in wheat grains, which may affect some rheological properties. Gao et al. (2018) studied the expression pattern of non-prolamins at specific growth stages under powdery mildew stress in five susceptible Chinese wheat cultivars and reported that globulin concentrations changed dynamically and significantly, especially at 25 days after anthesis when disease indices were the highest. However, the authors did not observe variations of grain albumin accumulation in any treatment with increasing powdery mildew severity.

#### Effect of Foliar Diseases Caused by Necrotrophs or Hemibiotrophs on Processing and End-Use Quality of Wheat

A different effect of necrotrophic pathogens with respect to biotrophics has been reported. An early report of effects of the necrotroph *P. tritici-repentis* causing increases in GPC (Rees et al., 1982) was confirmed by later results (Fleitas et al., 2018a; Castro et al., 2018; Figure 7). Conversely, *Z. tritici* usually either had no effect or increased GPC and/or gluten concentration (Gooding et al., 1994; McKendry et al., 1995; Puppala et al., 1998; Ishikawa et al., 2001; Dimmock and Gooding, 2002c; Ruske et al., 2003; Gooding, 2007; Blandino and Reyneri, 2009; Rodrigo et al., 2015; Castro and Simón, 2016, 2017; Fleitas et al., 2017; Castro et al., 2018; MacLean et al., 2018). However, there are situations when *Z. tritici* reduced GPC. For instance, Arabi et al. (2007) found that GPC reductions caused by *Z. tritici* depended on the susceptibility of the genotype with no GPC changes in resistant cultivars but significant decreases for the susceptible genotypes. Differences in the magnitude of modifications in the N/carbohydrates ratio in the different experiments probably cause these discrepancies. But in addition, reports where the GPC increased by the effect of *Z. tritici* suggest that the necrotroph stage of this pathogen is the main contributor for these effects or the lack of effect of this pathogen on the host growth during the asymptomatic phase as was reported by Sánchez-Vallet et al. (2015).

Nitrogen and fungicide treatments may show combined effects with respect to GPC. Ruske et al. (2003) found that, in some cases, reductions in GPC due to applications of fungicides to control *Z. tritici* were compensated by applications of foliar urea at anthesis. Penny et al. (1978) found a positive complementary effect between fungicide and liquid N applications at ear emergence on GPC under *S. nodorum* and *B. graminis* epidemics. However, Kelley (1993) reported that only under topdress N supply, GPC increased and foliar fungicide had no effect. Reductions of GPC for *Z. tritici* with triazole + strobilurin fungicides tended to decrease as N increased, whereas for inoculations with *P. tritici-repentis* no differences in GPC were evident among fungicide and untreated plots at the highest N rates. Conversely, under lower N rates, GPC decreased when fungicides were applied, mainly with the triazole + strobilurin + carboxamide treatment (Castro et al., 2018). Under high N rates, although fungicides may reduce grain shriveling due to a higher carbohydrates accumulation in the grains, N uptake and N translocation to the grains may generate this lower reduction in GPC.

Regarding the effect of necrotrophic and hemibiotrophic pathogens on rheological properties, results are not consistent. In the particular case of hemibiotrophic pathogens, the results are expected to be not as consistent or as marked as those observed for a pure necrotrophic organism because of the first biotrophic-like behavior. Therefore, responses between a necrotrophic and biotrophic pathogen can be expected (Figure 7). Thus, increases or no effect of *Z. tritici* in AlvW with the use of fungicides containing triazole-strobilurin have been reported. Discrepancies were also found for the AlvL and the AlvP/L ratio (Cátedra-Cerón and Solís Martel, 2003; Blandino and Reyneri, 2009; Castro and Simón, 2017). Differences between experiments may be partly due to the effect of several pathogens affecting bread-making quality at the same time under natural infections, in addition to the different bread-making aptitude of the genotypes. It has been reported that GPC increases in those cultivars with high bread-making quality (i.e., suitable for leavened-bread production) but tends to decrease in those cultivars with poor bread-making quality (Dimmock and Gooding, 2002c; Castro and Simón, 2017). The protein quality (gliadin/glutenin ratio) is essentially determined by the genotype. That is why positive conditions for the deposition of proteins and increments in the gluten concentration in “modern” varieties do not always correspond to AlvW increases or other gains in bread-making quality. Although GPC and gluten concentration can be estimated reciprocally, none of these parameters showed a significant association with AlvW (García et al., 2001). For instance, when GPC increased in cultivars with high bread-making aptitude, the AlvW improved, while in cultivars of lower bread-making aptitude, the AlvW values increased more slowly (Renzi et al., 2007).

In addition, the effect of pathogens in the gliadins/glutenins ratio may play a role in these discrepancies. It is likely that, when no alteration of rheological properties by specific pathogens was observed, protein composition was not modified. It has been demonstrated that in some cases, despite modifications in GPC, protein composition is not altered (Arabi et al., 2007). The effects of the diseases in the duration of phases in which gliadins and glutenins are formed is probably involved in these apparent contradictions. As stated previously, gliadins are the first storage proteins accumulated in the grain, while glutenins are synthesized later in the grain-filling stage. Under stressed conditions, glutenin synthesis and accumulation are the first protein fraction being interrupted, whereas gliadins generally continue. Therefore, the final gliadin/glutenin ratio in the grain changes, which causes weak glutens and doughs with less development time (Blumenthal et al., 1993). As other pathogens *Z. tritici* infections reduce the grain-filling development (Dimmock and Gooding, 2002b; Pepler et al., 2006); hence there might be proportionally more gliadins, as glutenins are the most affected and the gliadin/glutenin ratio increases, affecting the rheological properties (Stone and Savin, 1999). Related to this, Castro and Simón (2017) found that cultivars with the lowest flag leaf green area duration showed the highest decrease in AlvP and the highest AlvL, AlvW, and loaf volume values when affected by *Z. tritici*, which can be associated with a high gliadin/glutenin ratio.

## General Discussion: Challenges and Areas for Further Research

In addition to the need to increase the wheat yield to meet world demand, the international market also has increasing quality requirements for the industry, and milling and end-use quality are essential to determine its worth in the market. In many countries, this value is determined according to standards based on milling properties and GPC. In addition, dough rheological properties determining end-use quality are of crucial importance for millers. The increases in grain yield in recent years have caused a reduction in the end-use quality, which is determined genotypically but also affected by abiotic and biotic stresses. Although there is much information about abiotic stresses determining N dynamics, milling characteristics, and end-use quality, the incidence of biotic stresses, among them those caused by foliar fungal diseases, is less understood, and results found in the literature are sometimes inconsistent. This review critically summarizes the available information on the subject, discussing fundamental findings and concepts, interactions among diseases with N fertilization schemes, and fungicide applications. Controversial results, possible explanations through ecophysiological models, drawbacks and gaps in the information available, and further research needed to minimize the impact of the foliar diseases on wheat quality are also discussed.

Several important foliar diseases affect wheat worldwide. Some controversial results of the impact of such diseases on wheat quality are partly due to the different nutritional habits of the pathogens involved and their interactions with N fertilization. This review summarizes findings of the incidence of several foliar fungal diseases on milling and end-use quality. Three pathogens that are among the most important and for which there is recent information available were taken as representative of biotrophic (*P. triticina*), necrotrophic (*P. tritici*-*repentis*), and hemibiotrophic (*Z. tritici*). In recent years, the impact of these diseases on ecophysiological variables affecting yield and end-use quality has been addressed.

The effect of foliar wheat diseases on GPC and end-use quality depends on several factors, among them the intensity and growth stage when diseases develop. The effect of N availability on the severity of the diseases with different nutritional habits has shown controversial results. As mentioned previously, while it is generally accepted that the severity of biotrophic pathogens increases under high N rates (Jensen and Munk, 1997; Hoffland et al., 2000), the severity of necrotrophic pathogens shows a variable response with a tendency to decrease (Hoffland et al., 2000; Long et al., 2000; Carignano et al., 2008), and for hemibiotrophic pathogens such as *Z. tritici*, an increase (Gheorghies, 1974; Simón et al., 2002) or decrease (Johnston et al., 1979). Several factors, such as N availability in the soil, weather conditions that can promote the growth of the crop or the pathogen in a differential way, the type and amount of N applied, and specific N concentration for a given pathogen can modify these results. Furthermore, experiments under natural infections do not allow to discriminate pathogens precisely. In addition, the severity of the diseases may be different and could have been assessed in different ways when comparing different studies. Experiments considering only the area under the disease progress curve caused by the pathogens and its relation to yield, GPC, and end-use quality do not provide information on the absolute size of the canopy. This canopy may vary among different genotypes, sites, and seasons, and the remaining healthy tissue could be of different magnitude across experiments. Ecophysiological approaches assessing the HAD as was done in the joint experiments presented in this review are more appropriate to quantify GPC modifications. In addition, quantification of variations in GPC and end-use quality related to source/sink ratio and thousand kernel weight will help elucidate some questions.

As indicated, in recent years, joint inoculation experiments with several pathogens inoculated separately at the same time, with different N rates and fungicide types under defined N availability, allowed us to elucidate some of the questions (Castro et al., 2018; Fleitas et al., 2018a,b). These kinds of experiments are extremely laborious as many (and large) plots need to be inoculated and assessed. However, they are able to avoid differences in environments, the inoculated pathogen is dominant, and the genotypes and N availability in the soil are fixed. Those experiments also enable us to explore how pathogens of different nutritional habit impact differentially on crop growth at different growth stages. Despite their limitations, attempts to carry out more inoculated experiments assessing the effect of individual pathogens in different environments is necessary. The use of some recent specific fungicides that act mainly on biotrophic or on necrotrophic pathogens will be useful to better discriminate the prevalent pathogens when specific inoculations are carried out.

In fact, there are carboxamides that control primarily rusts whereas other carboxamides better control necrotrophic pathogens. In this sense, it has been reported that carboxamides such as benzovindiflupyr are more effective against leaf rust epidemics in comparison to another carboxamide molecules, such as fluxapyroxad and pydiflumetofen, which are widely used against necrothroph pathogens. Although scientific reports about this behavior are scarce (Gosling, 2018) there is already a commercial company promoting a fungicide with benzovindiflupyr and fluxapyroxad for specific control of rust and necrotrophic foliar diseases, respectively. These specific fungicides, used separately, would be able to better discriminate different types of pathogens in research experiments, although for wheat production it is usual to use fungicide brands composed of different modes of action. This allows expanding the control of pathogens and reducing the possibility of resistance development. In recent years more experiments have been carried out using ecophysiological approaches to analyze the impact of foliar diseases on wheat growth according to its nutritional habit (Serrago et al., 2009; Schierenbeck et al., 2016) and its consequences in yield and/or N dynamics and end-use quality (Castro and Simón, 2017; Fleitas et al., 2018a,b; Schierenbeck et al., 2019a).

The effect of foliar diseases on N dynamics, milling, and end-use quality depends not only on the intensity and nutritional habit of the prevalent pathogen but also on the time of infection, consequently determining whether the number of grains per unit area (early infection) or the thousand kernel weight (late infection) is most affected. As mentioned previously, how the relationship between carbohydrates and N is modified and how the crop is able to compensate for those modifications is critical for determining milling, processing, and end-use quality in wheat.

In addition, the effects of foliar diseases on protein composition have not been investigated. Aside from the effects on the GPC, the gliadin/glutenin ratio could affect the impact of the diseases on the rheological properties. Cultivars with the lowest flag leaf green area duration showed the highest decrease of AlvP and the highest AlvL, AlvW, and loaf volume values (Castro and Simón, 2017) when affected by *Z. tritici*, which can be related to the gliadin/glutenin ratio. As mentioned previously, these proteins accumulate in the grain asynchronously influencing dough rheological properties (Stone and Savin, 1999) and ultimately bread-making quality. More research needs to be done in this area.

Further research is also necessary on how a low source/sink ratio or a lower translocation of reserve stored on the stems can affect the reduction of thousand kernel weight or test weight, increasing grain protein and gluten concentration under late epidemics. A high source/sink ratio could reduce the use of soluble carbohydrates stored, whereas a low ratio could increase its translocation (Serrago et al., 2011). Experiments should be expanded to different environments and genotypes under infections with foliar pathogens with different nutritional habit.

In addition, although it has been demonstrated that soluble carbohydrates represent about 40% of dry matter in the stems at the beginning of the grain-filling stage (Reynolds et al., 2009), differences have been observed on its contribution to the grain yield (Foulkes et al., 2007; del Pozo et al., 2016) as its translocation can be affected by abiotic and biotic factors in a differential way. Genotypes have been found with a different concentration of soluble carbohydrates in the stems (Dreccer et al., 2009) which might exhibit a higher tolerance to foliar diseases during the critical period, although this hypothesis has not been confirmed. However, these genotypes may reduce the GPC due to a higher thousand kernel weight (Rozo Ortega, 2019).

Another area that warrants further research is how foliar diseases and fungicides to control them affect HFN. Large genotypic variability is observed in this variable in preliminary studies, and how genetic and environmental factors can influence its value has been studied. However, although it is known that fungicides can increase this problem affecting the baking industry, the physiology behind this phenomenon has not been investigated and should be addressed.

The end-use quality of each genotype will also determine how the pathogens will affect it, whereas the type of fungicide applied and the N fertilization rate could reverse negative effects caused mainly by biotrophic pathogens. Farmers should take into account the nutritional habits of the prevalent diseases to take management actions not only to increase grain yield but also end-use quality.

## Author Contributions

MSi prepared the first draft. MSi, MF, AC, and MSc wrote and reviewed the manuscript. All authors contributed to the article and approved the submitted version.

## Conflict of Interest

The authors declare that the research was conducted in the absence of any commercial or financial relationships that could be construed as a potential conflict of interest.
